# Escape from fraught states in a coordination game

**DOI:** 10.1098/rsos.231314

**Published:** 2024-02-21

**Authors:** Whitney Tabor, Garrett Smith, Harry Dankowicz

**Affiliations:** ^1^ Department of Psychological Sciences, U-1020, University of Connecticut, Storrs, CT 06269-1020, USA; ^2^ Department of Mechanical Engineering, University of Maryland, College Park, MD 20742-3035, USA; ^3^ Department of Mechanical Science and Engineering, University of Illinois at Urbana-Champaign, 1206 W. Green St. MC 244, Urbana, IL 61801, USA

**Keywords:** polarization, self-organization, Slider Game, agent-based model, consensus formation, local feedback

## Abstract

Through a behavioural coordination game played by groups of humans and simulated with agent-based models, we investigated a social network dilemma that we call *fraughtness*. Seven players, connected to one another in various topologies via a computer network, each had to move a slider to the left or right along a horizontal bar on their screen. The goal was for all the players to move their slider to the same side. Players received feedback indicating the degree to which they and their neighbours agreed about the choice of side. When the topology had a hierarchical branching structure, the groups often got stuck in fraughtness: players on one branch favoured one side, while players on the other branch favoured the other; because all were receiving supportive local feedback, nobody wanted to change. Nevertheless, after being stuck in fraughtness for some time, most groups managed to escape it. Fraughtness is arguably an analog of generally negatively viewed social phenomena like polarization and echo chambers. Our analyses suggest that while fraughtness is problematic, it is closely linked to successful structure formation—it thus may be most effective to focus not on how to banish it, but on how to resolve it.

## 1. Introduction

Human collectives often exhibit clustering—people affiliate with groups, typically aligning with like-minded and/or physically similar others. Several terms in the literature (e.g. ‘clique’, ‘polarization’, ‘echo chamber’) point to extreme and deleterious forms of such homophilic tendency. Corresponding research generally strives to understand its causes, often with an eye to avoiding or discouraging it. This perspective holds across a number of naturalistic [1–3], experimental [4–6], computational and/or mathematical [7–11], and review [12,13] studies.

Being also concerned about the socially deleterious effects of polarization, we nevertheless take a contrasting approach to those just mentioned. We note that cluster formation is an integral feature of many self-organizing systems, i.e. systems that consist of multiple independently acting but interacting entities that exhibit organized structure at the scale of the group [14]. In such systems, cluster formation is not easy to avoid and completely preventing it would likely eliminate the systems’ useful structure-forming abilities. At the same time, it is problematic when a group that needs to achieve coordination gets stuck in a polarization gridlock. Therefore, using a combination of human experiments and computational models, we studied how groups that fall into strong polarity sometimes manage to escape it. Relatedly, Li *et al*. [9] studied models in which polarization dissolves via a bifurcation (i.e. the change comes about through a parameter change). Also relatedly, [15–17] studied interventions that reduced polarization in both models and human behaviour. By contrast, in our models, the dynamics at a fixed parameter setting can enter into protracted polarization, which eventually dissolves spontaneously.

We examined a virtual coordination game that we designed, called the Slider Game. A number of players (seven in the groups we study here) are positioned at computer terminals, each displaying a horizontal slider bar. Each player can move the slider to the left or the right along this bar by pressing the arrow keys. The group is told that the goal of the game is for everybody to position their slider on the same side (either all fully to the left or all fully to the right). The players cannot see each other’s screens, but the computers are organized into a network with a particular topology, and each player sees a continuously evolving number that indicates the degree of positional alignment (called ‘Agreement’) among their topological neighbours. The game is designed in such a way that consensus is achieved if and only if each player reports 100% Agreement.

For the Slider Game, we formally define a collective property called *fraughtness*. Intuitively, fraughtness is a mixed state (some players’ sliders on the left side of the screen, some on the right) where each player is getting supportive local feedback from their neighbours so that they resist change. Fraughtness is thus a self-reinforcing form of polarization. While prior experimental [18–20] and computational [21–24] work on consensus formation has often focused on whether or not the groups converge and on what schedule, here we focus specifically on escape from fraughtness.^1^

We report four findings along with a theoretical interpretation. (i) Focusing on a particular branching topology with self-connections, we found that players fell into fraughtness at a relatively high rate, much higher than would be expected if the model were randomly transitioning between states. (ii) Despite often falling into fraughtness and persisting in this state for some time, most groups managed to escape fraughtness and achieve consensus before running out of time. (iii) The corresponding fully connected topology on seven players has no fraught states; nevertheless, we found some evidence that groups playing on this topology took longer to achieve consensus than groups playing on the branching topology. (iv) An averaging analysis of the slider position time histories showed a characteristic profile, which robustly appeared across distinct batches of runs. (v) We investigated several computational models, one of which produced an averaging profile similar to that of the humans. This optimal model supports a speculation that Agreement is akin to energy in a stochastic physical system; fraughtness is a form of metastability and groups escape it by waiting long enough for noise to dislodge the group from a higher-energy state into a lower one. In the model, the possibility of doing this depends on the model parameters being set within a narrow range of values.

## 2. Formal definition of agreement and fraughtness

Consider a network of players in a connected topology, in which every player is self-connected, and associate to the *i*th player a scalar, *s*_*i*_, called the player’s *position*, in the range [−1, 1]. At any point in time during the Slider Game, the quantity *s*_*i*_ represents the fractional displacement of the slider of the *i*th player from the centre of the slider bar toward the right (positive) or left (negative) end, with −1 corresponding to the leftmost position and 1 corresponding to the rightmost. Let $N_{i}$ denote the set of indices identifying player *i*’s neighbours, including *i*, and let $\left|N_{i}\right|$ equal the number of these neighbours.

**Definition 2.1 (Agreement).**

Given a position vector $s\;:=(s_{1},\ldots ,s_{N})$, the magnitude of the average position on the neighbourhood of the *i*th player,

2.1 $$A_{i}(s)\;:\;=\frac{\left|\Sigma_{\;j\in N_{i}}s_{j}\right|}{\left|N_{i}\right|},$$

is the *agreement* observed by the player.

It follows from the definition that $A_{i}(s)\in [0,1]$ for every s in the hypercube [−1, 1]^*N*^ and *i* = 1, …, *N*. Maximal agreement is observed by all players at the two diametrically opposite *consensus corners* {−1}^*N*^ and {1}^*N*^. We denote the orthants containing these corners by $O_{-}$ and $O_{+}$, respectively.

As noted in §1, it is the appearance of the value *A*_*i*_ on each player’s screen that allows the players to coordinate. We adopted this feedback format in alignment with many empirically supported models of animal (including human) swarming, which employ topologically governed averaging [26–28], motivated by data indicating that humans can make insightful decisions in swarms [29], and in keeping with data suggesting that human political opinions may be influenced by (average) opinions of their social groups as well as by rational analysis of facts and their implications [16,30].

Consider a position vector s such that no player’s position equals 0. For the *i*th player, define $F_{i}$ (the ‘friends of *i*’) as the set of indices of *i*’s neighbours, not including *i*, whose positions have the same sign as *s*_*i*_, and $E_{i}$ (the ‘enemies of *i*’) as the set of indices of those neighbours whose positions have the opposite sign. Let $F_{i}\;:=|\Sigma_{\;j\in F_{i}}s_{j}|$ and $E_{i}\;:=|\Sigma_{\;j\in E_{i}}s_{j}|$.

**Definition 2.2 (Fraughtness).**

The network is said to be in a *fraught state* if $s\notin O_{+},O_{-}$ and, moreover, *s*_*i*_ ≠ 0 and *F*_*i*_ ≥ *E*_*i*_ for *i* = 1, …, *N*.

Fraught states can be intuitively understood as the non-Pareto optimal Nash equilibria of the network agreement landscape under a sign selection strategy. Indeed, as shown in electronic supplementary material: *Properties*, for any state in the interior of an orthant with *F*_*i*_ ≥ *E*_*i*_ for *i* = 1, …, *N*, no player can increase their observed agreement by unilaterally switching the sign of their position, whereas such an increase must result for the *j*th player if *F*_*j*_ < *E*_*j*_. Moreover, *F*_*i*_ ≥ *E*_*i*_ for *i* = 1, …, *N* is trivially true for every state $s\in O_{+},O_{-}$. Fraught states, therefore, are those outside of the orthants $O_{+}$ and $O_{-}$ for which no player can increase their observed agreement by unilaterally switching the sign of their position. If we define the *strategy* of each player as the sign of their position and the agreement observed by the player as their *payoff*, then a fraught state is one with heterogeneous strategies in which no player can increase their payoff by unilaterally switching strategy. On the other hand, relative to the payoff in a fraught state, the payoff for some players must increase and for no player will decrease if all players choose the same strategy. In this sense, the fraught states provide a formal model of entrenched states of polarization among the players that are suboptimal in terms of agreement. As shown in the experimental data and reflected also in model simulations described in later sections, escaping such states is not impossible but requires coordinated action by the players such that several players temporarily suffer a loss of agreement. It was the hunch that some groups of human players might manage to achieve such delicate transitions despite the challenges involved that motivated us to explore this game design.

**Figure 1.**
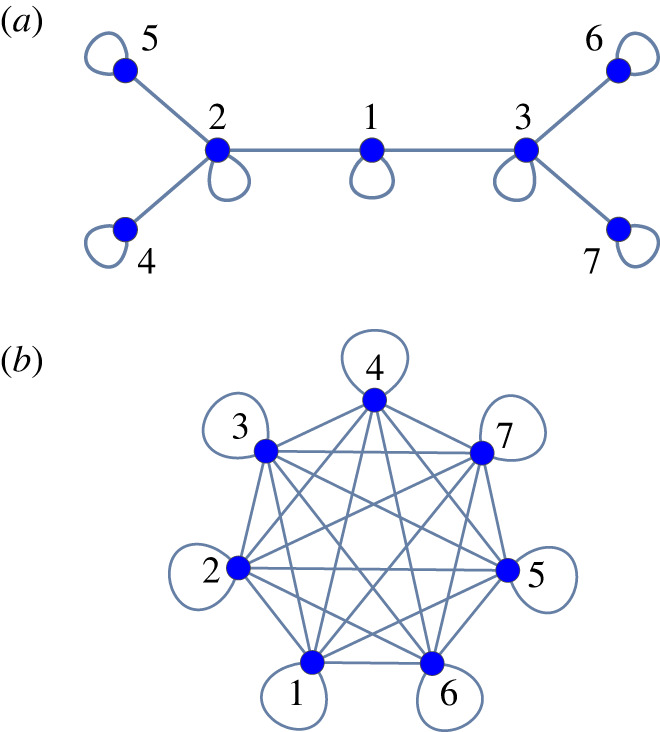
Topologies used in all experiments: (*a*) Hierarchy and (*b*) Full.

**Figure 2.**
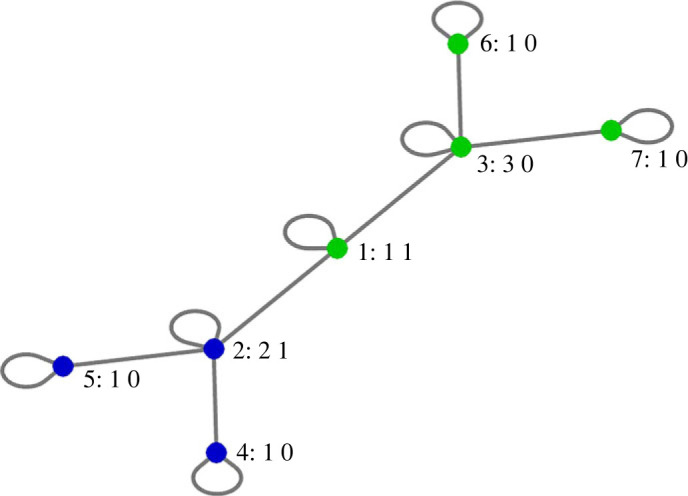
A fraught state for Hierarchy. Blue players have positions at −1. Green players have positions at +1. Each player *i* is labelled with three numbers: *i*: $F_{i}\;\;E_{i}$. Since $s\notin O_{-},O_{+}$, no player’s position equals 0 and, for every player, *F*_*i*_ ≥ *E*_*i*_, the network is in a fraught state.

In this work, we focus on the two topologies shown in figure 1 and referred to below as Hierarchy and Full. An example of a fraught state of Hierarchy is shown in figure 2. As shown in electronic supplementary material: *Properties*, regions of fraught states of Hierarchy only occur in a subset of orthants and comprise approximately 1.30% of the volume of the hypercube [−1, 1]^*N*^, whereas the Full topology has no fraught states.

## 3. Experiment overview

Since this project began in an exploratory vein, we collected an initial dataset to develop hypotheses, and we used later-collected datasets to test these. The first two datasets were collected in a laboratory of networked computers at the University of Connecticut. Participants were recruited through the Psychological Sciences Department participant pool and received course credit for their participation. The participants in the third (cloud-based) dataset were Amazon Mechanical Turk (‘MTurk’) workers who were paid for their participation. For all participants, the experimental protocol was approved by the Institutional Review Boards of the University of Connecticut and the University of Illinois at Urbana-Champaign. To gain insight into possible mechanisms underlying the patterns observed, we created three computational models. One of these showed many properties in common with the humans. Here, we focus on the Confirmatory Lab (i.e. second) Data Set and the best model. Details on the other datasets and models are given in electronic supplementary material: *Data Sets* and electronic supplementary material: *Models*. The data are available at https://osf.io/gvcpz/.

## 4. Human experiments

### 4.1. Method

#### 4.1.1. Procedure

For the laboratory-based experiments, seven players sat at separate computer terminals spaced at least two terminals apart in a large classroom with 18 or 20 desktop computers. Talking was not allowed. Each screen exhibited a horizontal slider bar, with a slider that could be incrementally moved to the left or the right using arrow keys. One key press moved the slider 1/20th of the distance between the left and right ends of the slider bar at a maximum speed of one full crossing in 2 s. Players could achieve the maximum speed by holding down one or the other arrow key. A group of at least two, and often 3 or 4 laboratory research assistants (RAs) ran each session. Before starting the game, they told participants that the goal was for all of the players to move their sliders to the same end of the slider bar. They explained that each player was connected to some subset of the other players and that each player would see on their screen a per cent value called ‘Agreement’ that would continuously indicate how well they were doing at coordinating with their subgroup (Agreement was computed as in equation (2.1) in terms of the fractional displacement *s_i_* of each slider from the center of the slider bar toward the right (positive) or left (negative) end). The round would terminate once every player’s Agreement reached 100% (corresponding to either of the two consensus corners of the [−1, 1]^7^ hypercube) or after a fixed time (5 min), whichever came first. The RAs also cautioned the players that the computer randomized what counted as ‘left’ and ‘right’ separately for each player on every round, so it would not be useful to look at other players’ screens to achieve coordination. An equivalent procedure applied to the cloud-based experiments except that the time limit was 3 min.

For a round to terminate as a result of every player’s Agreement reaching 100%, a group would need to break the symmetry between the two consensus corners and arrive at a choice of one side or the other emergently, through feedback interactions mediated solely via the displayed Agreement values. By randomly switching the assignment of ‘left’ and ‘right’ for each player between rounds, we both discouraged spying and reduced the likelihood that groups would establish conventions (e.g. ‘Always go to the left’) across rounds that could quickly allow them to converge on a solution.

#### 4.1.2. Materials

In addition to the Hierarchy and Full topologies (figure 1), which we focus on here, the experiments also included some other topologies. In the Exploratory Lab Data Set, 17 groups of seven players each attempted nine rounds (three Hierarchy, three Connected-Terminal Hierarchy^2^ and three Full). In the Confirmatory Lab Data Set, 21 groups of seven players each attempted nine rounds (three Hierarchy, three Ring^3^ and three Full). In the Confirmatory Cloud Data Set, 26 groups of seven players each attempted nine rounds (three Hierarchy, three Full and three Star.^4^) We have so far carefully analysed only the Full and Hierarchical datasets, but we plan to explore the Ring, Star and Connected-Terminal Hierarchy datasets in future studies.

#### 4.1.3. Predictions

Given the design of the Slider Game, we believed it plausible to assume that players generally get a strong feedback signal from changes in their observed Agreement that stem from their own actions and a much weaker signal from effects that their actions have on the actions of other players. Assuming then that each player moves their slider to maximize Agreement, such actions would be driven mainly by the sensitivity $\partial A_{i}(s)/\partial s_{i}$, when defined. As shown in electronic supplementary material: *Properties*, if fraught corners of the hypercube [−1, 1]^7^ were to exist, they would be locally attractive under the flow $\Phi_{A}$ of the self-gradient vector field

4.1 $${\;f}_{A}(s)=\left(\frac{\partial A_{1}(s)}{\partial s_{1}},\ldots ,\frac{\partial A_{N}(s)}{\partial s_{N}}\right),$$

and, consequently, limit points for open sets of initial conditions. Given such initial conditions, groups would become trapped in a state of fraughtness until one or several players, out of impatience, give up on single-mindedly trying to increase their Agreement and, instead, take actions that would allow the group to eventually escape. By the same analysis in electronic supplementary material: *Properties*, we found that the only other attractive limit points of $\Phi_{A}$ were the consensus corners, making it implausible that groups would become trapped in suboptimal states anywhere other than at fraught corners. We also thought it plausible that players would use their episodic memory to recall their Agreement when their sliders were on the opposite end of the slider bar; this knowledge could support coordination by providing a rational basis for remaining at or abandoning particular end points of the slider bar. Additionally, we anticipated a contrast between the Hierarchy and Full topologies. Because the latter has no fraught corners in which to get trapped, whereas each of the corners in the orthants in table 1 are fraught states for the former, we expected that convergence to either consensus corner would be more frequent and come more quickly in Full than in Hierarchy.

**Table 1.** Sign combinations representing orthants containing fraught states for the Hierarchy topology in figure 1*a*.

*s*_1_	*s*_2_	*s*_3_	*s*_4_	*s*_5_	*s*_6_	*s*_7_
+	−	+	−	−	+	+
−	−	+	−	−	+	+
+	+	−	+	+	−	−
−	+	−	+	+	−	−

### 4.2. Results

#### 4.2.1. Sample round

**Figure 3.**
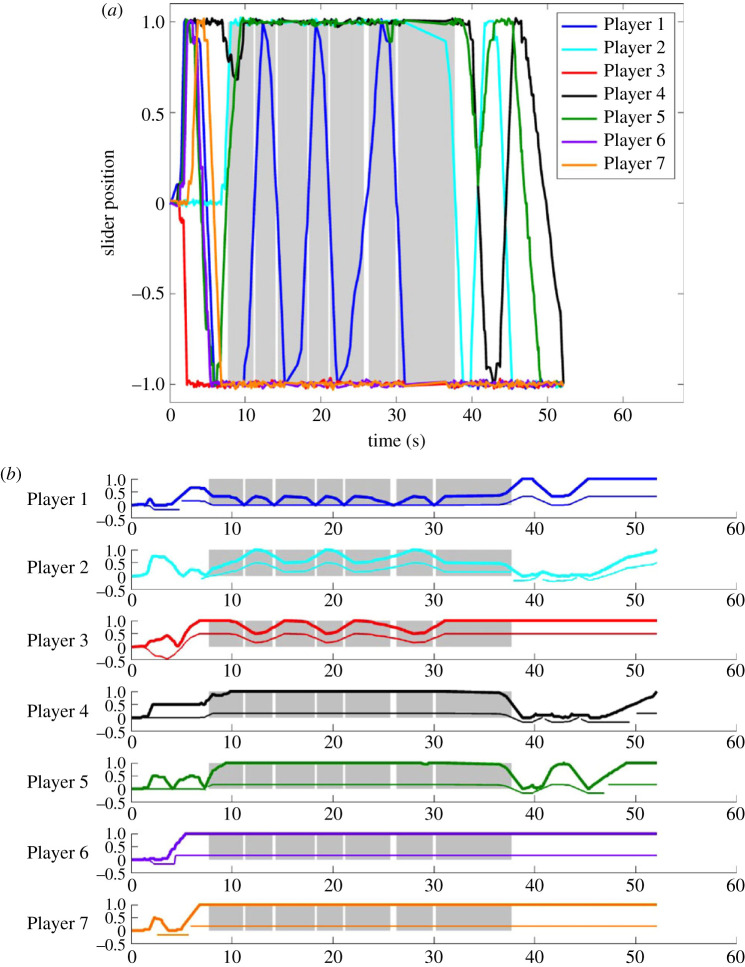
Time histories for (*a*) slider position *s*_*i*_ (jittered slightly to avoid overplotting); (*b*) agreement *A*_*i*_ (thick curves) and agreement asymmetry *F*_*i*_ − *E*_*i*_ (thin curves, scaled for legibility) for each of the seven players in a typical substantially fraught round on Hierarchy per the numbering in figure 1*a*. In the shaded regions, $s\notin O_{-},O_{+}$, and *s*_*i*_ ≠ 0 and *F*_*i*_ > *E*_*i*_ for *i* = 1, …, 7—i.e. the state is fraught. The players are numbered the same way in figures 1*a*, 2 and 3.

Figure 3*a* shows the slider position *s*_*i*_ of each of seven players versus time for a typical round on Hierarchy from the Confirmatory Lab Data Set exhibiting an extended period of fraughtness. Figure 3*b* shows the Agreement *A*_*i*_ associated with each player during the round shown in figure 3*a*, along with the corresponding *agreement asymmetry* *F*_*i*_ − *E*_*i*_. This run illustrates a case where the network was not initially in a fraught state; then fell into fraughtness with the player at node 1 displaying approximately oscillatory behaviour for a while; then escaped fraughtness and eventually reached a consensus corner.

#### 4.2.2. Basic statistics

Each session with the 21 groups in the Confirmatory Lab Data Set included three versions of Hierarchy, three versions of Full and three versions of Ring, with the versions ordered pseudorandomly within each group. We labelled a round as *successful*, if the group reached consensus within the 5 min time limit.

Putting Ring aside, we considered only the successful rounds and asked if there was any effect of *topology* or *presentation position* on the time until consensus. For the performance comparison between Hierarchy and Full, using data from 21 groups of seven participants each, the Hierarchy topology was faster on average (*M* = 61.61 s, s.d. = 52.97 s) than Full (*M* = 87.01 s, s.d. = 62.04 s). We analysed the log-transformed times until consensus with a mixed effects model in R using the lme4 package [31]. The model included fixed effects for presentation position (ranging from 0 to 8) and topology, as well as by-group random intercepts. Both main effects were significant (*p*′*s* < 0.05). The presentation position effect showed that game duration got shorter as the experiment went on, suggesting an overall learning effect (*b* = −0.08, s.e. = 0.03, *t* = −2.38). Regarding the effect of topology, Hierarchy was significantly faster than Full (*b* = −0.39, s.e. = 0.16, *t* = −2.50), indicating that the above-reported difference in means is significant. A similar comparison of Hierarchy and Full from the Confirmatory Cloud Data Set revealed a difference in means that went in the same direction, but the result was not significant (*b* = −0.24, s.e. = 0.13, *p* = 0.07). These results largely contradict our prediction that Full would be faster than Hierarchy—we return to this point in §7.

On average, 11.6% of each Hierarchy round was spent in fraughtness. As fraught regions comprise approximately 1.30% of the volume of the position space, they appear to have an attracting character, at least over intermediate time scales. A histogram of per cent fraughtness values by round showed a clear divide between two clusters of rounds: one with fraughtness rates close to 0% and the other with fraughtness rates over 10%. We therefore defined ‘substantially fraught’ as greater than 10%. There were 19 substantially fraught rounds (out of 63 rounds of Hierarchy in total). Considering just these 19 rounds, the average fraughtness was 36.4%. Despite the potential challenges of this high rate of fraughtness, 16 of the 19 rounds succeeded in escaping fraughtness and reaching consensus before running out of time.

Overall, these results suggest that, with the Hierarchy topology, although fraughtness is a liability, the groups are fairly efficient at escaping fraught states. One of our main aims in this work was to understand how groups fall into fraughtness and how they escape it. In the next section, we report the results of an averaging analysis, which highlights persistent features of this process.

#### 4.2.3. Averaging analysis

**Figure 4.**
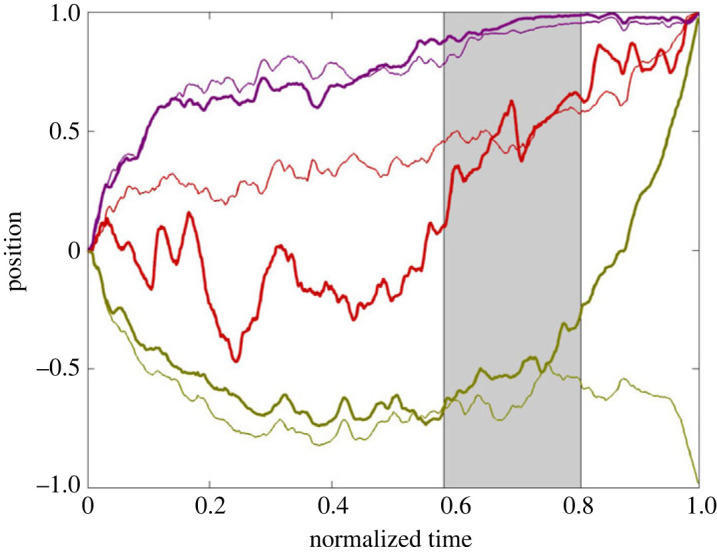
Averaged normalized trajectories from 16 successful rounds on the Hierarchy topology with at least 10% of time spent in fraught regions from the Confirmatory Lab Data Set. Thick curves: averages of the Left Branch (green), Right Branch (purple) and Apex (red). Thin curves: average agreement values for the corresponding branches—for the Left Branch, the sign has been flipped for ease of readability. The shading shows the Agreement Adverse (see electronic supplementary material: *Methods*). Electronic supplementary material: *Data Sets* includes versions of this figure that are additionally shaded to portray variance.

For the Hierarchy topology, we call nodes {2, 4, 5} the *Left Branch*, nodes {3, 6, 7} the *Right Branch* and node 1 the *Apex*. Figure 4 shows an average of all successful but substantially fraught trajectories from the Hierarchy rounds of the Confirmatory Lab Data Set.^5^ To make this figure, we first rasterized all rounds so they had the same number (1500) of equally spaced time steps and then divided discrete time by 1500 so it ranged from 0 to 1. Moreover, if necessary, the sign of each player’s position was flipped *a posteriori* so that every dataset converged on the consensus corner {1}^7^. Additionally, if needed, the identity of the ‘Left’ and ‘Right’ branches was flipped *a posteriori*, so the average position of the Right Branch was greater than the average position of the Left Branch over the whole round. This last step aligns the groups with respect to their branch biases. Note that, in figure 4, both branches have an approximately monotonically changing derivative—the Right Branch is concave down and the Left Branch is concave up. The thin lines show the average agreement value of each group. For ease of readability, the Left Branch thin line shows the negative of Left Branch agreement.

Based on the exploratory analysis, which produced a similar figure to figure 4 (see electronic supplementary material: *Data Sets*), we identified a number of formal properties of the average graphs that might be relevant to the question of how groups fall into, and then later escape, fraughtness. These properties are listed in table 2. Indeed, the average curves for the Confirmatory Lab Data Set (figure 4) as well as for the Confirmatory Cloud Data Set (electronic supplementary material: *Data Sets*, figure S3*a*,*b*) both exhibit the properties listed in table 2, thus providing support for the thesis that the properties identified by the exploratory analysis are systematic consequences of the design of the Slider Game. A question arises, though, as to which properties stem from inherent characteristics of the game and which tell us something about the nature of the players’ decision-making processes—see §6 below.

**Table 2.** Features derived from the exploratory analysis of the average trajectory of groups on Hierarchy that pass through extended periods of fraughtness (cf. figure 4).

(a) The Left Branch trajectory has a quadratic form that is concave up, while the Right Branch has a rise-to-asymptote structure such that during the middle of the normalized trajectory, the average behaviour is substantially fraught.
(b) The Left Branch trajectory can be divided into the following stages:
(i) descent into fraughtness: position and average agreement progress together
(ii) sitting in fraughtness: static low position and high average agreement
(iii) climbing laboriously out of fraughtness: average agreement decreases for a time (the ‘Agreement Adverse’)
(iv) final rush to convergence with low variability: rapidly regaining average agreement
(c) The Right Branch trajectory can be divided into the following stages:
(i) an initial steep climb into fraughtness: position and average agreement progress together
(ii) a gradual increase thereafter to convergence: position and average agreement progress together
(d) The Apex’s position, though noisy, stays near zero during approximately phases (i) and (ii) of the Left Branch, with corresponding agreement value at around 33%. Approximately coincident with phases (iii) and (iv) of the Left Branch, the Apex’s position rises to 1 and its agreement rises to 100%.

**Figure 5.**
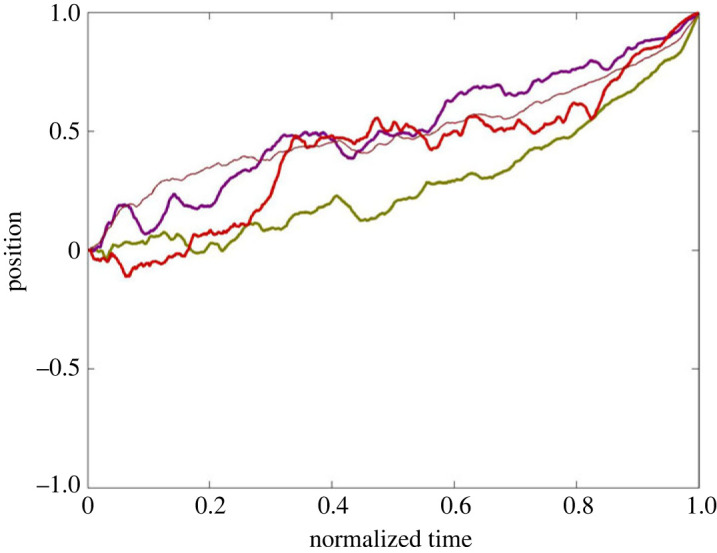
Averaged normalized trajectories for successful rounds on the Full topology for ‘Left Branch’ (green), ‘Right Branch’ (purple) and ‘Apex’ (red). Note that the terms ‘Left Branch’, ‘Right Branch’ and ‘Apex’ have no meaning in the Full topology; they are just arbitrary subgroups of players. Agreement, which has the same value for all seven players, is shown by the thin curve.

As a test of our assumptions, we applied the same averaging method to the Full topology runs from the Confirmatory Lab Data Set (figure 5). In the Full topology, if players are optimizing agreement per the self-gradient flow $\Phi_{A}$, then only consensus corners are attractive. In this case, there is no topological significance to the terms ‘Left’ and ‘Right’. As expected, the so-called ‘Right Branch’ is consistently above the so-called ‘Left Branch’ because we picked the designations ‘Left’ and ‘Right’ in the same way as we did in the analysis of Hierarchy described above, checking to see which subgroup, on average, was closer to 1. However, also as expected, there is no evidence of plateauing with the groups on opposite sides of the slider bar.

## 5. Discussion of human experiments

The averaging analysis provides some confirmation of our expectation that the humans in the Hierarchy topology might escape fraughtness in a systematic way. The agreement trajectories are particularly noteworthy: although players generally act in such a way as to increase agreement over time, after the system has been stuck in fraughtness for some time, the Left Branch undergoes a notable decrease in agreement during its phase (iii) when it is escaping from fraughtness. It seems as though the disposition of the Left Branch players toward their agreement values is adjusted during this phase. We refer to this decreasing agreement phase as the *Agreement Adverse* (see caption of figure 4).

To better understand the implications of the results of the human experiments, we made several computational models. We discuss these next.

## 6. Simulation experiments

### 6.1. Models

To help clarify which features of figure 4 reveal properties of the players, which stem from properties of the game, and which are artifacts of the analysis, we implemented several agent-based models of the Slider Game on the Hierarchy topology, as detailed in electronic supplementary material: *Models*.

In the simplest model (Random Walk or ‘RW’), model agents execute independent random walks on [−1, 1] according to some predetermined step size, Δ. As it appeared impractical to have such a model produce a significant number of runs that both reached consensus and exhibited substantial (more than 10%) fraughtness for small Δ, we set Δ = 2, so that agents jumped randomly from −1 to 1 and back. This resulted in a profile very different from figure 4. Among other things, the average agreement for all branches (not just the Apex) hovered at or below one-third for the bulk of each run and the concavity of the Right Branch did not match the human data. Moreover, as can be surmised without simulation, the overall rate of fraughtness was close to the inherent fraughtness volume (1.30%), corresponding to the fact that in this model, the fraught regions have no particular attractive power.

A natural, more sophisticated hypothesis holds that players follow the agreement gradient by detecting whether their movement in a particular direction increases or decreases observed agreement and choosing the direction of increase. As observed in §2, for a simple, self-gradient-following scheme, fraught corners are attractive limit points. Thus, in the second candidate model (Gradient Following with Random Switches or ‘GFRS’), we let the direction of travel be determined by the self-gradient with agents making randomly triggered jumps to the other side of the slider bar in order to escape fraughtness. This model produced a better approximation of the human data than RW, but still fell short on several counts—the concavity of the Right Branch was distinct from that of the human data; and the random switching mechanism is inaccurate with respect to the human dynamics, for the humans are not able to make big jumps in position.

To avoid such unrealistic jumps in position, in a third model (Agreement Position Emigration or ‘APEM’), agents move at a constant rate (when possible) along a direction that only changes for a randomly selected agent after some randomly chosen time, provided that the absolute value of their position exceeds a *position threshold*, $s_{\theta }$, and their observed agreement is below an *agreement cut-off*, $a_{\theta }$. This model is meant to implement a natural combination of assumptions about what drives human behaviour in the Slider Game:

(i) Humans are arguably goal-oriented; rather than continuously responding to forces that impinge on them from moment to moment (as, for example, a molecule does), they tend to select an action and carry it out before deciding whether to select a subsequent action; to emulate this, an agent in the model selects a side of the slider and heads more or less steadily towards it.
(ii) Human behaviour is modulated by an attentional mechanism, which randomly fluctuates in sustained tasks [32]; the model therefore implements human goal-directedness as intermittent attention: an agent sets its sights on a goal (getting to one end or the other of the slider), sets out on the relevant course and does not consider deviating from this course until its attention is randomly drawn to the question again.
(iii) The position/agreement criteria for switching sides are motivated by principles of the causal nature of actions: if one has just begun to execute an action (e.g. if one has only gone a little way toward switching the side of ones’s slider), then one is not yet in a strong position to evaluate the suitability of the choice to switch—hence the positional criterion mentioned above. On the other hand, once one has completely carried out an action, if the desired effect is still far from being achieved (corresponding to having a low agreement value), then one would do well to consider taking a different action—hence the agreement criterion mentioned above.

APEM also includes a speed parameter, Δ, which specifies the maximum speed at which agents can traverse the slider, a time limit parameter, *K*, corresponding to the maximal time each group is given to reach consensus, and an attentional rate parameter, *μ*, corresponding to the mean time interval between successive instances of an agent evaluating the position/agreement criterion for their slider. We report next on results of simulations of APEM and refer the reader to electronic supplementary material: *Models* for typical trajectories for RW and GFRS.

### 6.2. Simulation results

In the notation of electronic supplementary material: *Models*, we let Δ = 1, *K* = 300, *μ* = 0.1 and $a_{\theta }=s_{\theta }=0.5$. Here, the values of Δ and *K* are consistent with the maximal traversal rate and time limit in the lab experiments (one full crossing in 2 s and 5 min, respectively). Regarding the values chosen for *μ*, $a_{\theta }$ and $s_{\theta }$, we provide motivations below.

**Figure 6.**
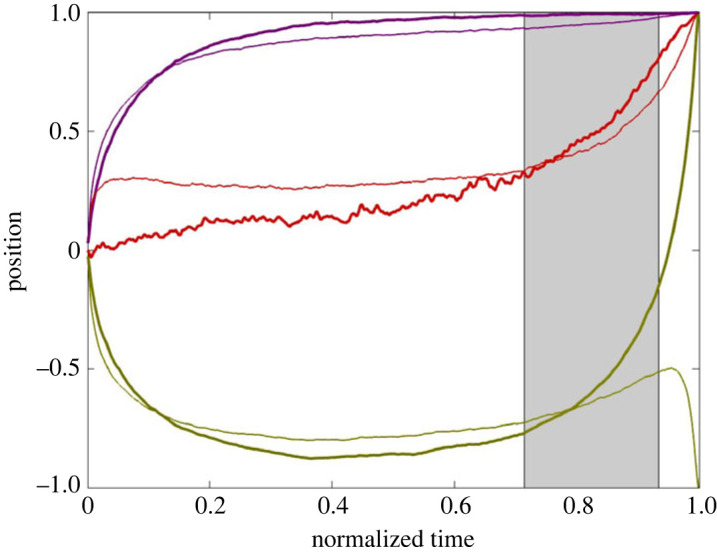
Averaged normalized trajectories of position (thick lines) and agreement (thin lines) generated from 1000 successful runs of the APEM model with more than 10% fraughtness for the Left Branch (green), Right Branch (purple) and Apex (red). The thin curve for the Left Branch is the negative of its agreement (cf. figure 4).

Figure 6 shows the average of 1000 model simulations on the Hierarchy topology that reached consensus before running out of time and satisfied the same fraughtness criterion (greater than 10% per run) used when analysing the human data. While the corresponding human result (figure 4) was based on only 16 game runs, we included many more in the model analysis so its average behaviour can be easily discerned. With regard to the properties of the human branch profiles outlined in table 2 for Hierarchy, the APEM model gets every property correct, while, as noted above, the other two models each fail on at least one count. For this reason, and also because the APEM model’s assumptions better match the cognitive mechanisms that people plausibly use to solve this task, we prefer the APEM model.

We performed a corresponding test of the APEM model on the Full topology and found that its profile resembled the human data in figure 5 inasmuch as neither the humans nor the model show any signs of fraughtness, and the magnitude relationships among the curves as well as the variance properties are similar between model and humans. However, the shapes of the APEM trajectories are quite nonlinear—see electronic supplementary material: *Models*—while those of the human trajectories look almost linear. This discrepancy may reflect how humans are able to use their memory of recent gameplay to incrementally progress toward a solution in Full—a possibility we leave for future investigation.

**Figure 7.**
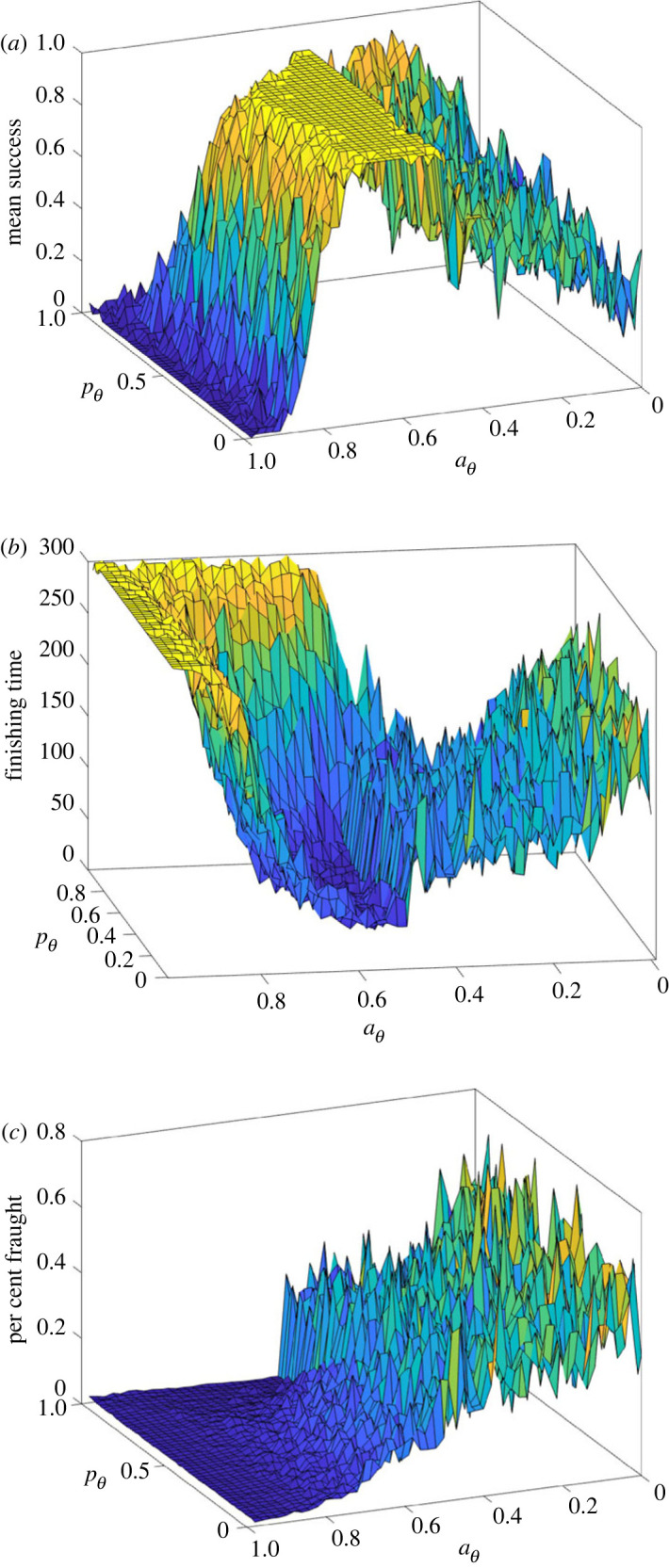
(*a*) Parameter space map of the APEM model showing success rate (within the 5 min time limit) as a function of the agreement cut-off ($a_{\theta }$) and the position threshold ($s_{\theta }$); (*b*) time to consensus; (*c*) per cent fraughtness per round. Brighter colours (e.g. yellow) indicate high values, while darker colours (e.g. blue) indicate low values on the vertical axes.

We explored a range of possible values for the four model parameters $a_{\theta }$, $s_{\theta }$, *μ* and *K*. For the model to reach consensus, *K* (the game time limit) has to be large enough that the agents have time to detect and respond to each other’s choices; additionally *μ* (the mean of the distribution of time delays) must be substantially smaller than *K* to avoid fixity; interestingly, if *μ* is very small, the model tends to wallow in fraughtness for long intervals, so to achieve high rates of success, *K* must be increased accordingly. With *K* set to match the human game lengths (300) and *μ* set to a compatible value (0.1), figure 7*a* shows the percentage of runs that reach consensus before running out of time, and figure 7*b* shows average values of the time until consensus across the range of possible values of $a_{\theta }$ and $s_{\theta }$. For maximizing the likelihood of consensus and minimizing the time until consensus, there is a precise optimum: $a_{\theta }=0.5$ and $s_{\theta }=0.5$. Figure 7*c* shows that, for parameter values near this choice, fraughtness is present at a low-intermediate level (10–20%), consistent with the human data (≈11.6%). Interestingly, from a human’s point of view, it makes intuitive sense to switch sides if one’s position is more than halfway toward an extreme ($s_{\theta }=0.5$) and agreement is less than halfway to the optimum ($s_{\theta }=0.5$). Motivated by these observations, we set the parameters in the above simulation to the values just mentioned (see electronic supplementary material: *Models*).

### 6.3. Discussion of simulation experiments

Several points from the simulation results stand out. The APEM model produces average outputs that are qualitatively consistent with the profile from averaging of the human Hierarchy data, even though the former assumes agents with a fixed, Markovian character (i.e. they lack both episodic memory and the ability to adjust their parametrization). This finding does not rule out the possibility that humans use their episodic memories or undergo a dispositional shift based on experience that helps them succeed at the task, but it warns against prematurely assuming that they do so. Interestingly, all the models (including RW) show some degree of Agreement Adverse in connection with departure from fraughtness. This suggests that the Agreement Adverse stems more from the structure of the game than it does from the particular method employed by the players. At the same time, the correspondence between APEM and the humans, along with the ability of both to exit fraught states after they have thoroughly gone into them, suggests that we might learn something about how humans can address fraughtness by analysing the model. We consider this point further in §7.

## 7. General discussion

Based on our experience with the Slider Game, we suggest that there is utility in investigating human group behaviour in contexts where the interactions can be fully and precisely measured, and which are, at the same time, formal settings that can be linked to existing insights about collective behaviour [33–35].

In a series of experiments, we asked networked human participants or simulated agents to reach consensus in a binary-choice game by varying their positions along a continuum between two extreme values in response to local feedback constrained by the network topology. The topology had a surprising effect on how participants were able to solve the task: groups playing on a branching topology had to contend with periods of fraughtness that trapped the groups in suboptimal metastable equilibria of positional alignment before eventually escaping and reaching consensus. By contrast, groups playing on a fully connected topology, which does not support fraughtness, took longer to reach consensus. We attribute the delay in consensus to a difference in signal-to-noise ratio where players in the fully connected topology were less able than those in the branching topology to ascertain the effect of their own contributions on the local feedback signal. This result is one motivation for our initial claim that it may be counterproductive to try to completely expunge societal homophily.

Although it is not our purpose in this paper to fully explore predictions of the APEM model, we do intend to set the stage for such exploration. An anonymous reviewer pointed out that it is natural to ask whether the escape-from-fraughtness pattern observed here scales up to larger groups. Preliminary investigation of randomly generated tree-shaped networks with 40–60 agents and low maximum degree showed a U-shaped fraughtness profile roughly similar to the pattern we have observed here with Hierarchy, suggesting that fraughtness can be an issue for somewhat larger networks as well. Future research should further explore scaling up.

As we have noted, the APEM model findings urge caution in ascribing the groups’ ability to escape fraughtness to complex mental mechanisms in humans like frustration-tendency and episodic memory. Why, then, do the groups reliably go through a phase of decreasing the Left Branch Agreement on the way to escaping fraughtness? We make a speculative observation. Agreement is roughly analogous to (the negative of) energy in a spin glass or ferromagnet. In an Ising model (simple physics model of such materials), the component of Hamiltonian energy, *H*_*i*_, associated with one electron, *i*, in a lattice, absent an external field, is

7.1 $$H_{i}=-\Sigma_{\;j\in N_{i}}s_{i}s_{j}=-s_{i}\;\Sigma_{\;j\in N_{i}}s_{j},$$

where *s*_*i*_, an electron spin, takes its values in {−1, 1}. Such materials can discover a low-energy (highly aligned) state via annealing—starting at a high temperature, the temperature is lowered on a schedule that causes the system to occupy a series of local energy minima (metastable states) on the way to reaching a global minimum. Perturbations induced by kinetic energy (non-zero temperature) allow the system to broach energy barriers that would otherwise block it from reaching lower-energy states. Let $S_{i}=\Sigma_{\;j\in N_{i}}s_{j}$. On the region (*s*_*i*_, *S*_*i*_) ∈ [−1, 1] × [−|$N_{i}$|, |$N_{i}$|], there are two minima of *H*_*i*_, located at (*s*_*i*_, *S*_*i*_) = (−1, −|$N_{i}$|) and (*s*_*i*_, *S*_*i*_) = (1, |$N_{i}$|). These are the same as the locations of the maxima of *A*_*i*_ on the region of player *i*’s interaction with their neighbours in the Slider Game. The random time sampling of APEM introduces a noise element that we suggest plays a role analogous to the role of kinetic energy in the Ising model, supporting the broaching of low-agreement barriers—we speculate that such events are the source of Agreement Adverses. This interpretation of APEM dynamics is consistent with the evidence reported in [15–17] suggesting that conceptual dissonance can drive belief change. We noted that efficient escape from fraughtness in APEM only occurs within a limited range of parameter settings. Observationally, in both the model and the human groups, the property that confers efficiency is the propensity to go rapidly and thoroughly into a structured regime, effectively plumbing its qualities, and then, if it proves lacking, to exit it. At this point, we do not know why or how the humans seem to naturally have their parameters set in a way that causes them to do this, but we think the question is worthy of further investigation.

To conclude, we note that there is much to be done in exploring the effects of topology on Slider Game coordination dynamics. So far, we have only considered fixed topologies but, as an anonymous reviewer pointed out, it will be of interest to adopt a plausible mechanism for letting players modulate their connections in response to what is happening in the game and determining whether fraughtness still arises and plays the same central role in game outcomes that we have seen here. Finally, we note that several of the properties we have derived here concerning the existence and stability of fraught states rely on the assumption of self-connectedness and would not carry over to networks on simple graphs, which is a condition that may also be worthy of exploration.

## Data Availability

The data are available at https://osf.io/gvcpz/. Supplementary material is available online [36].

## Footnotes

1 We came to this work via the study of an artificial system that has an unstable equilibrium of zero agreement [25], which nevertheless attracts an open set of initial conditions, suggesting the possibility of entrapment followed by escape.

2 Connected-Terminal Hierarchy is the same as Hierarchy except that it has two additional connections: 4 linked to 5, and 6 linked to 7 (cf. figure 1*a*).

3 In the Ring topology, all players are self-connected and also connected to one another in a single loop.

4 In the Star topology, all players are self-connected, and a single central player is connected to every other player.

5 We included multiple instances from the same group (three instances of Hierarchy were run for each group) in order to produce a smoother average. A possible worry is that the form of the global average is an artifact of the combination of early and late rounds for each group. To allay this concern, we also examined averages of just the first instance of Hierarchy, just the second and just the third from each group, using a more relaxed cut-off for inclusion in the analysis (more than 5% rather than greater than 10% fraught). These plots were noisier than the global average with the stricter cut-off reported in the main text, but each of the three analyses showed a qualitative structure largely aligned with the description in table 2.

## References

[1] Cinelli M, Morales GDF, Galeazzi A, Quattrociocchi W, Starnini M. 2021 The echo chamber effect on social media. Proc. Natl Acad. Sci. USA **118**, e2023301118. (10.1073/pnas.2023301118) 33622786 PMC7936330

[2] Morales AJ, Dong X, Bar-Yam Y, Pentland AS. 2019 Segregation and polarization in urban areas. R. Soc. Open Sci. **6**, 190573. (10.1098/rsos.190573) 31824692 PMC6837204

[3] Neal ZP. 2020 A sign of the times? Weak and strong polarization in the U.S. Congress, 1973–2016. Soc. Netw. **60**, 103-112. (10.1016/j.socnet.2018.07.007)

[4] Eschert S, Simon B. 2019 Respect and political disagreement: can intergroup respect reduce the biased evaluation of outgroup arguments? PLoS ONE **14**, 1-12. (10.1371/journal.pone.0211556) PMC6435108 30913232

[5] Yahani E, Gallagher N, Merhout F, Cavalli N, Guilbeault D, Leng Y, Bail CA. 2022 An Online experiment during the 2020 US–Iran crisis shows that exposure to common enemies can increase political polarization. Sci. Rep. **12**, 19304. 36369344 10.1038/s41598-022-23673-0 PMC9652360

[6] Sieber J, Ziegler R. 2019 Group polarization revisited: a processing effort account. Pers. Soc. Psychol. Bull. **45**, 1482-1498. (10.1177/0146167219833389) 30885061 PMC6732819

[7] Coscia M, Rossi L. 2022 How minimizing conflicts could lead to polarization on social media: an agent-based model investigation. PLoS ONE **17**, 1-23. (10.1371/journal.pone.0263184) PMC8794152 35085365

[8] Kashima Y, Perfors A, Ferdinand V, Pattenden E. 2021 Ideology, communication and polarization. Phil. Trans. R. Soc. B **376**, 20200133. (10.1098/rstb.2020.0133) 33612005 PMC7935022

[9] Li L, Scaglione A, Swami A, Zhao Q. 2013 Consensus, polarization and clustering of opinions in social networks. IEEE J. Sel. Areas Commun. **31**, 1072-1083. (10.1109/JSAC.2013.130609)

[10] Wu Y, Li L, Yu Q, Gan J, Zhang Y. 2023 Strategies for reducing polarization in social networks. Chaos, Solitons and Fractals **167**, 113095. (10.1016/j.chaos.2022.113095)

[11] Yang VC, Abrams DM, Kernell G, Motter AE. 2020 Why are U.S. parties so polarized? A ‘satisficing’ dynamical model. SIAM Rev. **62**, 646-657. (10.1137/19M1254246)

[12] Hewstone M, Rubin M, Willis H. 2002 Intergroup bias. Annu. Rev. Psychol. **53**, 575-604. (10.1146/annurev.psych.53.100901.135109) 11752497

[13] McPherson M, Smith-Lovin L, Cook JM. 2001 Birds of a feather: homophily in social networks. Annu. Rev. Sociol. **27**, 415-444. (10.1146/annurev.soc.27.1.415)

[14] Haken H. 2008 Self-organization. Scholarpedia **3**, 1401. (10.4249/scholarpedia.1401)

[15] Dalege J, der Does TV. 2022 Using a cognitive network model of moral and social beliefs to explain belief change. Sci. Adv. **8**, eabm0137. (10.1126/sciadv.abm0137) 35984886 PMC9390990

[16] Galesic M, Olsson H, Stein DL. 2021 Integrating social and cognitive aspects of belief dynamics: towards a unifying framework. J. R. Soc. Interface **18**, 20200857. (10.1098/rsif.2020.0857) 33726541 PMC8086875

[17] van der Does T, Stein DL, Fedoroff N, Galesic M. 2023 Science communication in light of moral and social concerns: testing a statistical physics model of belief change. OSF preprints https://osf.io/zs7dq/.

[18] Kearns M. 2010 Experiments in social computation. Commun. ACM **107**, 14 978-14 982.

[19] Kearns M, Suri S, Montfort N. 2006 An experimental study of the coloring problem on human subject networks. Science **313**, 824-827. (10.1126/science.1127207) 16902134

[20] Judd S, Kearns M, Vorobeychik Y. 2010 Behavioral dynamics and influence in networked coloring and consensus. Proc. Natl Acad. Sci. USA **107**, 14 978-14 982. (10.1073/pnas.1001280107) PMC2930545 20696936

[21] Boyer D, Miramontes O. 2003 Interface motion and pinning in small-world networks. Phys. Rev. E **67**, 035102. (10.1103/PhysRevE.67.035102) 12689120

[22] Castellano C, Vilone D, Vespignani A. 2003 Incomplete ordering of the voter model on small-world networks. Europhys. Lett. **63**, 153-158. (10.1209/epl/i2003-00490-0)

[23] Castellano C, Loretto V, Barrat A, Cecconi F, Parisi D. 2005 Comparison of voter and Glauber ordering dynamics on networks. Phys. Rev. E **71**, 066107. (10.1103/PhysRevE.71.066107) 16089820

[24] Dall’Asta L, Baronchelli A, Barrat A, Loreto V. 2006 Nonequilibirium dynamics of language games on complex networks. Phys. Rev. E **74**, 036105. (10.1103/PhysRevE.74.036105) 17025706

[25] Saghafi M, Dankowicz H, Tabor W. 2017 Emergent task differentiation on network filters. SIAM J. Appl. Dyn. Syst. **16**, 1686-1709. (10.1137/16M1084432)

[26] Bialek W, Cavagna A, Giardina I, Mora T, Silvestri E, Viale M, Walczak AM. 2012 Statistical mechanics for natural flocks of birds. Proc. Natl Acad. Sci. USA **109**, 4786-4791. (10.1073/pnas.1118633109) 22427355 PMC3324025

[27] Couzin I. 2018 Synchronization: the key to effective communication in animal collectives. Trends Cogn. Sci. **22**, 844-846. (10.1016/j.tics.2018.08.001) 30266143

[28] Helbing D, Johannson A. 2013 Pedestrian, crowd, and evacuation dynamics. (http://arxiv.org/abs/1309.1609)

[29] Rosenberg L, Wilcox G, Halafi S, Lungren M, Baltaxe D, Lyons M. 2018 Artificial swarm intelligence employed to amplify diagnostic accuracy in radiology. In *Proc. of the 9th Annual Information Technology, Electronics, and Mobile Communication Conf. (IEMCON)*, *Vancouver, BC, Canada*, pp. 1186–1191. IEEE. (10.1109/IEMCON.2018.8614883)

[30] Kahan DM, Peters E, Wittlin M, Slovic P, Ouellette LL, Braman D, Mandel G. 2012 The polarizing impact of science literacy and numeracy on perceived climate change risks. Nat. Climate Change Lett. **2**, 732-735. (10.1038/nclimate1547)

[31] Bates D, Mächler M, Bolker B, Walker S. 2015 Fitting linear mixed-effects models using lme4. J. Stat. Soft. 67, 1-48. (10.18637/jss.v067.i01)

[32] Esterman M, Rothlein D. 2019 Models of sustained attention. Curr. Opin. Cogn. Psychol. **29**, 174-180. (10.1016/j.copsyc.2019.03.005) 30986621

[33] Chacoma A, Zanette DH. 2015 Opinion formation by social influence: from experiments to modeling. PLoS ONE **10**, 1-16. (10.1371/journal.pone.0140406) PMC4627778 26517825

[34] Sobkowicz P. 2009 Modelling opinion formation with physics tools: call for closer link with reality. J. Artif. Soc. Soc. Simul. **12**, 11.

[35] Stewart AJ, Mosleh M, Diakonova M, Arechar AA, Rand DG, Plotkin JB. 2019 Information gerrymandering and undemocratic decisions. Nature **573**, 117-122. (10.1038/s41586-019-1507-6) 31485058

[36] Tabor W, Smith G, Dankowicz H. 2024 Escape from fraught states in a coordination game. Figshare. (10.6084/m9.figshare.c.7073592) PMC10878803 38384775

